# Mpox in South Asia: A Narrative Review on Epidemiology, Preparedness, and Preventive Strategies

**DOI:** 10.1002/hsr2.72249

**Published:** 2026-04-06

**Authors:** Jannatul Mawa, Shariun Nahar Rimun, Nujhat Zayma Rahman, Mohiuddin Ahmed Bhuiyan, Mohammad Shahriar, Ramisa Anjum

**Affiliations:** ^1^ Department of Pharmacy University of Asia Pacific Dhaka Bangladesh

**Keywords:** anti‐viral, control measures, epidemiology, Mpox, one health approach, South Asia, vaccination

## Abstract

**Background and Aims:**

Monkeypox (Mpox), due to its growing global concern, was declared a public health emergency of worldwide concern by the World Health Organization in August 2024. The monkeypox virus (MPXV), once limited to African regions, has now spread to South Asia, including Pakistan, India, Nepal, and Sri Lanka, and there have been over 109,699 cases and 234 deaths reported globally till now. This review aims to provide an overview of the spread of Mpox in South Asia, the preparedness of the South Asian countries, and the preventive and control measures.

**Methods:**

This narrative review was conducted through a comprehensive literature search of online databases including PubMed, Google Scholar, World Health Organization (WHO) resources, World Bank Open Data, and relevant public health reports from South Asian countries. Published literature was reviewed to extract information on mpox transmission, pathogenesis, epidemiology, preventive measures, healthcare preparedness, and regional impact.

**Results:**

Currently there is no clinical treatment for Mpox. Several antiviral agents are undergoing investigation, including cidofovir, brincidofovir, and so forth. In vitro potency of cidofovir for Mpox was found to be highest among other antivirals in a study. Tecovirimat did not significantly reduce the time required to heal Mpox lesions in two separate studies. In a different investigation involving trifluridine, tecovirimat, when used in combination, showed a substantially additive effect in dermal fibroblast cells (DF) and immortalized human conjunctival epithelial cells HConEpic. Alteration of biological traits, human behavior and clinical manifestations of Mpox mandates a need for surveillance of transmission. Regional outbreak patterns were identified through this study. Control measures include vaccination of susceptible individuals and avoiding contact with infected animals and humans.

**Conclusion:**

Mpox poses a growing public health threat in South Asia due to population density, limited healthcare infrastructure, and underreporting. Strengthening vaccination, antiviral management, and diagnostic capacity is crucial to control transmission. Tailored region‐specific strategies, including surveillance and stigma reduction, are essential.

## 1 Introduction

When the World Health Organization (WHO) declared Mpox as a Public Health Emergency of International Concern on August 14, 2024, it raised concern all over the world [1]. From January 1, 2022, to September 30, 2024, there were 109,699 confirmed cases of Mpox and 234 deaths caused by this infection reported worldwide across 123 countries [2, 3]. Mpox, which was formerly known as Monkeypox, is a highly infectious disease caused by the zoonotic monkeypox virus (MPXV) belonging to the Orthopoxvirus (OPXV) genus of the *Poxviridae* family [4, 5]. The first recorded case of monkeypox occurred in 1958 amongst captive *Macaca fascicularis* monkeys at Statens Serum Institut in Copenhagen, Denmark [4, 5]. In the same year, the term “monkeypox virus” was first introduced, and in November 2022, the WHO officially renamed it ‘Mpox’ [4]. The first documented human case of Mpox occurred in 1970 in the Democratic Republic of Congo in a 9‐month‐old infant [4, 6]. However, a recent investigation showed evidence of this virus in historical samples of the skin of African rope squirrels (*Funisciurus* sp.) that were collected from all over Central Africa over 120 years and kept in museums. This proves that Mpox has existed for well over 6 decades [4]. Mpox is a DNA virus that can be classified into Clade I and Clade II, which can be further classified into subclade IIa and IIb. In 2024, a newly identified subclade of Clade I called Clade Ib, was discovered in Africa [5].

The most common manifestations of MPXV infection are rash, fatigue, fever, and shivering. Some other symptoms include‐ itching, swollen lymph glands, joint pain, headache, myalgia, etc. These symptoms generally appear within 3 weeks of contact with the virus, and this infection usually persists for 2–4 weeks [7]. However, studies indicate that smallpox vaccines have been 85% effective in preventing monkeypox [6]. Healthcare workers, people who work with animals, and sex workers are at the highest risk of coming in contact with Mpox [7].

As of August 2024, South Asia has recorded a limited number of Mpox cases: 11 in Pakistan, 30 in India, 1 in Nepal, and 4 in Sri Lanka, with no cases reported in other countries [3]. During COVID‐19, close collaboration of federal agencies, academia and industry accelerated the approval of vaccines and therapies, leading to swift control of the pandemic. Since then, public awareness regarding infectious diseases and preventive measures have also increased. It is very important to learn from the past public health crises and avoid the mistakes made back then [8]. According to World Bank data, the majority of the South Asian countries fall under the category of Low‐ and Middle‐Income Countries (LMIC) [9]. Before COVID‐19, many LMICs relied on imports for medical tools, reagents and diagnostics. Due to border closures, it was extremely difficult for these countries to get access to the medical supplies required to combat COVID‐19. Therefore, LMICs require a stronger drug manufacturing capacity [10].

MPXV is structurally quite similar to smallpox. Therefore, even though no vaccine has specifically been approved for MPXV, smallpox vaccines can provide some immunity against MPXV infections. In order to reduce side effects from original smallpox vaccines and provide better protection against MPXV, some stabilized versions‐ ACAM2000, MVA‐BN, LC16‐ have been developed [11]. On December 16, 2024, Serum Institute of India (SII) announced that they have partnered with Bavarian Nordic A/S to transfer technology for the manufacturing of MPXV vaccine. This agreement allows SII to manufacture, sell, and distribute the vaccine in India after receiving the necessary regulatory approvals. This would increase global production of Mpox vaccines and their accessibility during future outbreaks [12].

Although the numbers are still low, the potential for swift spread remains a serious concern. This review article aims to analyze the regional outbreaks of Mpox in South Asia and assess the impact of this virus on these countries. It also discusses the prevention and control strategies. Furthermore, it also explores the threats that Mpox poses and the measures taken by South Asian countries to prevent the spread. Some limitations and areas of future research are also discussed in this review.

## 2 Methods

This study was conducted as a narrative review to provide a comprehensive overview of the epidemiology, transmission, pathogenesis, preparedness, and preventive strategies of monkeypox (Mpox) in South Asia. A literature search was performed using multiple electronic databases, including PubMed, Google Scholar, World Health Organization (WHO) reports, World Bank Open Data, and official public health publications from South Asian countries. In addition, relevant government reports and trusted regional news sources were reviewed to capture recent outbreak information. The search was conducted using combinations of keywords such as *“monkeypox,” “*Mpox,*” “South Asia,” “epidemiology,” “transmission,” “pathogenesis,” “preparedness,” “vaccination,”* and *“public health response.”* Articles published in English were included. Peer‐reviewed articles, surveillance reports, epidemiological updates, and authoritative institutional documents were considered. Studies focusing on non‐human orthopoxviruses or unrelated viral infections were excluded. The selected literature was narratively synthesized to summarize current knowledge regarding Mpox transmission dynamics, clinical manifestations, preventive measures, healthcare preparedness, and public health challenges in South Asia. The findings were organized thematically to provide a coherent overview of regional trends and response strategies.

## 3 Epidemiology of Mpox in South Asia

Since early 2022, Mpox cases have spread widely, with over 109,000 confirmed cases and 236 deaths reported in 123 countries across all WHO regions as of September 2024. A recent 8% increase in new cases shows that the virus continues to spread, especially in Africa, where 63.6% of cases were reported last month, followed by the Americas at 15.5%. Mpox has always been endemic in African regions, but this year has seen a sharp rise in the Democratic Republic of the Congo (DRC), with over 35,000 cases and 1006 fatalities reported. This increase, linked to viral strains clade 1a and clade 1b, signals a serious shift from the usual pattern. The United States has recorded the most cases worldwide (34,063), closely followed by Brazil and Spain. The top ten nations account for almost 79% of all cases worldwide, together with additional severely impacted nations including France, the UK, and Colombia [3].

Mpox has emerged as a significant issue in South Asia with varying case counts reported across the region. Since the first discovery in April 2023, 11 cases and one fatality have been recorded in Pakistan [1]. Since 2022, at least 32 incidents—including one fatality—have been documented in India. A new case of the highly transmissible clade 1b form was found in India in September 2024, which is associated to the ongoing Mpox outbreak in Africa [13, 14]. Till this date, Sri Lanka has recorded four cases, while Nepal has confirmed one [3]. No Mpox instances have been documented in other South Asian nations yet. The impact of Mpox in Sri Lanka must be interpreted within the broader context of ongoing and recent infectious disease challenges [15]. The country has previously experienced significant public health strain due to COVID‐19 [16], recurrent dengue outbreaks [17], and periodic influenza epidemics [18]. These concurrent viral diseases have placed sustained pressure on healthcare infrastructure, surveillance systems, and diagnostic capacity. In such settings, the emergence of Mpox represents an additional burden, particularly in terms of case detection, laboratory confirmation, and public risk communication. Social factors such as economic instability, population mobility, and limited zoonotic surveillance further complicate outbreak preparedness and response. While the number of reported cases of Mpox in South Asian countries remains relatively low compared to other regions, the potential for rapid transmission is alarming. This situation emphasizes the need for ongoing awareness and preventive actions throughout the region in order to properly manage possible epidemics.

**Figure 1:**
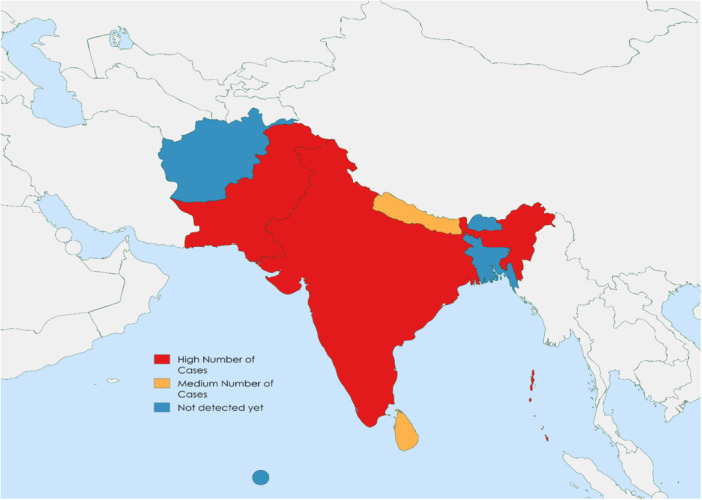
Distribution of Mpox cases in South Asia.

Figure 1 shows a map generated using WHO data, illustrating the distribution of Mpox cases in South Asia.

## 4 Disease Overview

### 4.1 Virology

MPXV, a member of the OPXV genus in the *Poxviridae* family, a DNA virus is the causative agent of monkeypox, a developing zoonotic disease. It is related to small pox virus. The reservoir of the virus and its natural cycle are yet to be known [19]. Mpox virus (MPXV) particles measure 200–250 nm and exhibit a brick‐shaped morphology. They feature a lipoprotein envelope enclosing a core with double‐stranded DNA (dsDNA) and lateral bodies. MPXV manifests in two forms: the single‐membrane mature virion (MV) and the double‐membrane extracellular enveloped virion (EV). Replication occurs in the host cell cytoplasm. The dsDNA genome spans approximately 197.2 kb, encoding 181 proteins, with covalently closed hairpin termini and ~10 kb inverted terminal repeats (ITRs) at both ends. Genes are densely packed, with intergenic regions rarely exceeding 100 bp [20, 21]. The virus can still exhibit two different morphologies: the mature virion (MV), which has a single membrane and the double‐membraned extracellular enveloped virion (EV) [21]. MPXV is endemic to several African nations and comprises two phylogenetic clades: the less virulent West African clade (case fatality rate ~ 4%) and the more pathogenic Congo Basin clade (case fatality rate ~ 10%), with the 2022 global outbreak linked to the former from a single unidentified source.

Central African strains show higher morbidity, mortality, human‐to‐human transmission, and viremia than West African ones [22].

### 4.2 Pathogenesis

Mpox is transmitted through direct contact with skin lesions, bodily fluids, respiratory droplets, and contaminated fomites. Following exposure, the virus enters through broken skin or mucous membranes, including ocular, oral, respiratory, urethral, and rectal surfaces. Epidemiological evidence from the current outbreak indicates that contact with skin lesions is the primary mode of transmission. After entry, the virus spreads to local tissues and lymph nodes via immune cells, constituting the incubation period, which may last up to 2 weeks and is typically asymptomatic [23, 24]. Following incubation, infected individuals may develop prodromal symptoms such as fever, chills, headache, myalgia, and lymphadenopathy, usually lasting up to 3 days. This phase is followed by the appearance of skin lesions that initially emerge on the upper body and subsequently disseminate. The rash progresses from papules to vesicles, pustules, and crust formation, with complete resolution occurring within 2–4 weeks. During the current outbreak, atypical clinical presentations have been reported, particularly among men who have sex with men (MSM), including initial perianal lesions. Severe complications such as septicemia, hemorrhagic disease, necrotic lesions, obstructive disease, and organ inflammation may occur in acute cases [23]. Mpox viral infection involves three major stages: invasion, replication, and assembly with release of progeny virions [23]. Similar to other poxviruses, MPXV infects a wide range of mammalian cells and replicates entirely within the host cell cytoplasm without reliance on specific cellular receptors [25]. Viral entry occurs through attachment, membrane fusion, and core release, either at the cell membrane or following endocytosis. Glycosaminoglycans, including heparin and chondroitin sulfates, facilitate viral attachment to host cells, while several viral envelope proteins play critical roles in membrane fusion and entry [25]. Following cytoplasmic replication, mature virions may remain intracellular or acquire additional envelopes to form extracellular virions responsible for systemic dissemination and cell‐to‐cell spread [26]. Although significant advances have been made in understanding MPXV replication, further research is required to fully elucidate the complexity of its cytoplasmic life cycle [25]. Mpox is generally a self‐limiting disease; however, disease severity varies depending on viral strain, host immune status, and associated complications [23].

## 5 Transmission Dynamics of MPXV

### 5.1 Animal to Human Transmission

MPXV is transmitted from animals to humans via a variety of mechanisms, the most common of which are percutaneous exposure, which involves direct contact with infected animals' broken skin; exposure of mucous membranes, such as those in the mouth and rectum; and inhalation of infectious particles in enclosed spaces where infected animals reside. Rodents (including Thomas's rope squirrels and Gambian pouched rats) are considered to be the principal reservoirs for MPXV. Evidence indicated that although the virus was first discovered in laboratory monkeys, it did not spread widely among primates. The 2003 outbreak in the United States was linked to imported mice from Ghana, highlighting the risk of zoonotic transmission and the need to track wildlife trade. Studies have shown that rodents often exhibit serological signs of infection; a notable study conducted in 1987 found that the seroprevalence of Thomas's rope squirrels in Zaire (now the Democratic Republic of the Congo) was 24.7%. Experiments have also shown that infected rodents can excrete significant amounts of MPXV in their saliva and feces, which could contaminate the environment and spread the virus to people. For example, Kellen's dormouse has shown a high vulnerability to infection, releasing the virus early in the disease and raising concerns about future transmission. To reduce the chance of animal‐to‐human transmission, public health activities should involve educating the public about the dangers of handling sick wildlife and eating bushmeat. While some animals, such as primates, may only be contagious when they have obvious lesions, rats can carry MPXV without exhibiting symptoms [27, 28, 29, 30, 31].

### 5.2 Human to Human Transmission

MPXV is transmitted from person to person in a variety of ways, including direct contact with infected individuals, respiratory droplets exchanged in close proximity, and fomite transmission through contaminated surfaces or items [32]. Recent outbreaks (particularly since 2022) have highlighted sexual contact—especially among gay, bisexual, men who have sex with men (GBMSM)—as a significant transmission mode, with many cases involving multiple or anonymous partners [33].

Emerging evidence emphasizes the dynamics of person‐to‐person transmission in various settings, particularly households. Nolen et al. demonstrated a higher likelihood of transmission among individuals sharing cups, dishes, and sleeping spaces, supporting the hypothesis of surface or fomite transmission; however, activities such as kissing and laundry did not correlate with virus acquisition. Close contact among inmates who shared communal spaces with a confirmed Mpox case in a Chicago prison facility did not result in transmission, even among those who received post‐exposure prophylaxis, indicating that transmission may be limited by shared hygiene practices and confined interactions [32, 34]. Healthcare workers remain at risk, as evidenced by documented cases of transmission despite the use of protective gear; Besombes et al. reported instances in the Central African Republic [32, 35, 36, 37], while Learned et al. identified familial transmission in the Democratic Republic of Congo. There are additionally reports of person‐to‐person transmission among children aged 7 months to 17 years, which could be attributed to contaminated tattoo and piercing materials or sexual transmission [38, 39, 40]. Vaughan et al. analyzed the epidemiological landscape across 36 European countries and concluded that sexual transmission was most likely the dominant cause of illnesses, but surface exposure and non‐sexual contact also had a role [41]. Notably, high virus levels were discovered on surfaces touched by infected individuals in four studies that investigated the presence of MPXV on surfaces in households, workplaces, and hospitals. Viable virus was detectable on contaminated surfaces for at least 15 days after contact, suggesting a risk of infection via contaminated surfaces [42, 43, 44, 45].

## 6 Prevention and Control Measures

### 6.1 Vaccines

High‐risk individuals, including laboratory technicians and healthcare professionals, should receive preventative Mpox vaccinations both before and after exposure, according to the Advisory Committee on Immunization Practices (ACIP). While post‐exposure prophylaxis (PEP) is crucial after unprotected exposure to infected individuals or contaminated materials, pre‐exposure prophylaxis (PrEP) aims to immunize patients before likely exposure. The CDC advises administering PEP vaccinations 4–14 days after exposure to effectively prevent Mpox infection. There are two FDA‐approved vaccines right now, which are JYNNEOS (MVA‐BN) and ACAM2000. For anyone in the US who is at risk of contracting Mpox, the CDC advises receiving two doses of the *JYNNEOS* vaccine. The non‐replicating JYNNEOS vaccine, which is made from modified vaccinia Ankara, offers around 85% protection against Mpox due to its safety profile. It is especially beneficial to persons with compromised immune systems and provides protection against both Mpox clades and all of their subclades. Despite its usefulness, ACAM2000 should not be used by those with impaired immune systems due to the risk of myocarditis and encephalitis [46, 47, 48, 49, 50].

Recent breakthroughs in the Mpox vaccine development process have yielded a plethora of intriguing choices. In animal studies, the *4pox DNA vaccine* provided considerable protection while targeting a diverse set of viral proteins. Further research is being conducted on multivalent mRNA vaccines, which have demonstrated efficacy in animal models and target multiple Mpox antigens. mRNA vaccines are making their way into human trials in China after demonstrating high efficacy in animal experiments. Vaccination initiatives are generally directed at high‐risk groups, such as males who have sex with other men, rather than the broader public. Protein‐based subunit vaccines with viral protein components are being developed; however, adjuvants are necessary for efficacy [48, 49].

Other noteworthy candidates include OrthopoxVac (VACΔ6), a live vaccine licensed in Russia with fewer adverse effects, and Vaccinia Virus Tian Tan (VTT), which has succeeded in smallpox prevention and is being adapted particularly for Mpox. KVAC103, derived from the Lister strain, has shown promise because of its safety and ability to induce protective immune responses. The NYVAC vaccine, based on the Copenhagen strain, is safe for some people, despite its decreased immunogenicity. Although there is less information on the effectiveness of the LC16m8 vaccine during Mpox outbreaks, it also shields animals from lethal dosages of viruses linked to Mpox. It uses a weakened virus and can spread to other body areas. Common side effects are fever, fatigue, and swollen lymph nodes. Over 90,000 people, including 50,000 children, have safely received LC16m8, though it's not recommended for immunosuppressed individuals, pregnant women, or people with specific skin conditions [48, 49, 50, 51].

Global efforts are also underway to develop subunit, peptide, and nucleic acid‐based vaccines targeting specific viral proteins known to induce strong protective immunity. Several additional vaccine candidates are being researched, including the Aventis Pasteur smallpox vaccine, which may be adapted for Mpox, and TNX‐801, which has shown promise in animal studies. The development of novel vaccinations against Mpox is another area of ongoing bioinformatics research, highlighting the necessity of creative strategies to successfully counter this new health risk [49, 50]. We have compiled some approved, potential and in‐research Mpox vaccines in Table 1.

**Table 1:** Mpox vaccine overview.

Vaccine name	Type	Status	Origin/Licensed Country	Reference
JYNNEOS	Live, non‐replicating vaccine	FDA approved	Denmark	[48, 49]
ACAM2000	Live virus vaccine	FDA approved	USA, Australia, and Singapore	[48, 52]
OrthopoxVac (VACΔ6)	Live virus vaccine	Potential candidate	Russia	[50, 53]
Vaccinia Virus Tian Tan (VTT)	Unattenuated, live virus vaccine	Potential Candidate	China	[48]
KVAC103	Attenuated, Live virus vaccine	Potential Candidate	Korea	[48]
NYVAC	Attenuated, Live virus vaccine	Potential Candidate	—	[48]
LC16m8	Attenuated, Live virus vaccine	In research	Japan	[48]
TNX‐801	Live virus vaccine	In research	—	[49, 50]
Aventis Pasteur smallpox vaccine	Live virus vaccine	In research	—	[49, 50]
*4pox DNA vaccine*	Nucleic acid vaccine	In research	—	[48]
Multivalent mRNA vaccines	Nucleic acid vaccine	In research	—	[48]
Protein‐based subunit vaccines	Subunit vaccine	In research	—	[48]

### 6.2 Antivirals and Therapy

Although the FDA has not approved any specific therapy for Mpox, many antiviral drugs, such as Cidofovir, Brincidofovir, and Tecovirimat, are currently used to treat the virus. These drugs were created to treat smallpox but are now used to control Mpox symptoms and prevent its spread. Cidofovir (Vistide) works by stopping viral DNA replication, although it may harm the kidneys. In a study conducted to compare the antiviral action of different agents on different poxviruses, cidofovir outperformed methisazone (141 μM) and ribavirin (106–238 μM) in terms of in vitro potency (IC₅₀ 47–78 μM)‐in case of MPXV [54]. Cell line‐dependent distinctions were highlighted by the fact that the antiviral effect was stronger in BSC cells than in Vero cells. Brincidofovir (CMX001), the prodrug of Cidofovir, is more readily available orally and presents fewer kidney risks, while it may affect liver enzymes. Tecovirimat (TPOXX) is said to reduce symptoms and mortality by targeting the viral protein and preventing virus release. Investigation regarding use of tecovirimat in treatment of infections caused by viruses related to smallpox virus is underway. In August and December 2024, results from initial analysis of data from the PALM007 and STOMP randomized clinical trials were made available. These trials were performed to evaluate the safety and effectiveness of a 14‐day course of tecovirimat in treating human Mpox. The results from both TPOXX and placebo recipients demonstrated that the antiviral medication did not shorten the time for Mpox lesions to resolve but it was safe to use [55]. Unfortunately, rapid rise in tecovirimat resistance has been reported in Mpox patients in the recent outbreak [56]. Another topical medication, Trifluridine (TFT), is used for treating eye infections caused by the Mpox virus. It works by inhibiting DNA synthesis and is usually safe, with mild side effects like eye irritation, but shouldn't be used for extended periods. A study of the efficacy of trifluridine (TFT) in treating Mpox infection in ophthalmic cells was performed. Assessment of infection rate of Mpox virus in two ophthalmic cell types: primary human keratocytes and immortalized human conjunctival epithelial cells (HConEpiC), was done in comparison to primary human dermal fibroblast cells (DF). The results showed the Mpox virus to cause a more pronounced cytopathogenic effect in epithelial cells compared to DF. Evaluation of antiviral efficacy of TFT on Mpox virus isolates from the 2022 outbreak was done in DF, keratocytes and HConEpiC. In all three types of cells, the dose of TFT inhibited all of the Mpox isolates dependently. Drug combinations with TFT were also assessed. When combined with brincidofovir, TFT showed a mild antagonistic effect on DF (Clwt: 1.25) and keratocytes (Clwt: 1.19). This combination had an almost additive impact in HConEpiC (Clwt: 0.92). While the combination of TFT and tecovirimat was shown to be somewhat antagonistic in keratocytes (Clwt: 1.29), it showed a substantially additive effect in DF (Clwt: 0.9) and HConEpic (Clwt: 0.94). The use of brincidofovir and tecovirimat together in DF was also examined. Additive effects against the Mpox virus were found, despite the fact that this combination has been shown to exhibit synergistic efficacy against the cowpox and vaccinia viruses [56]. Additionally, a passive immunotherapy called Vaccinia Immune Globulin (VIG) lowers viremia and mortality; however, further study is required to see whether it can be used specifically for Mpox [46, 49].

### 6.3 Control Measures

Healthcare and care workers who are at risk of exposure, people who have close contact with someone who has Mpox (such as family members, roommates, and caregivers), people who have multiple sex partners, including men who have sex with men, and sex workers of any gender and their clients are among the groups that may be at high risk of contracting Mpox. High‐risk individuals are also those who have HIV or other immunosuppressive diseases. Vaccination is a crucial preventive intervention for these groups and can be given after possible exposure (post‐exposure prophylaxis). The vaccine is most effective when administered after 4 days of exposure, but if symptoms have not appeared, it can still be administered up to 14 days later.

Current MPXV transmission patterns include shifts in the biological traits of the virus, human behavior, and clinical manifestations, making surveillance especially difficult in nations with limited resources. Although the death rate from the recent spread of MPXV clade II is rather low, young children and immunocompromised people—including HIV patients—are more vulnerable to severe consequences. Additionally, the spread of this virus has had a disproportionate impact on men who have sex with men. This suggests that sexual networks are a major source of transmission. This emphasizes how crucial it is to take into account several pathways of transmission when conducting Mpox surveillance and taking measures for prevention [57].

Disease surveillance data are a must to identify possible outbreaks and stop them before they become public health problems. To address the surveillance challenges a study on a community‐based surveillance system to detect Mpox by the utilization of used condoms was done. 20,941 condoms were collected from 16 South Asian, Southeast Asian and African countries. Out of 20,941 collected samples, 262 (1.3%) tested positive for DNA of MPXV. Highest positivity rates were found in India‐ 32/1188 (2.7%), Pakistan‐ 30/1302 (2.3%) and Thailand‐ 26/1256 (2.1%). Lowest positivity rates were found in Nepal‐ 8/1397 (0.6%), Papua New Guinea‐ 11/1429 (0.8%) and Maldives: 11/1429 (0.8%). All the samples contained clade IIb MPVX DNA. Regional outbreaks were indicated by the frequent close grouping of samples from the same or nearby countries. This investigational study proved to be an effective surveillance tool for Mpox transmission [57]. Thus, such tools can be used for surveillance purposes.

Most people with Mpox will recover within 2–4 weeks. However, to prevent transmission to others, individuals should take several measures [46, 47, 58, 59]:

1. Staying away from animals that could carry the virus, like primates and rodents.
2. Cooking animal meat thoroughly and wash it well before eating.
3. Quarantining infected animals properly to stop them from spreading the virus to other animals.
4. Avoid touching things that may have come into contact with infected animals or people.
5. Be cautious when physically interacting with others, especially if they're showing symptoms.
6. Washing your hands often and sanitizing is one of the simplest and most effective ways to protect yourself, especially after touching potentially contaminated things, and using protective gear when you're around infected individuals.
7. Isolating infected people to keep the virus from spreading.
8. Cleaning and disinfecting any areas where an infected person have been and hospital floors.
9. Refrain from sex during periods of high transmission, and use condoms for up to 12 weeks after recovery to protect yourself and others.
10. Healthcare workers should wear proper PPE like gloves, gowns, masks, and eye protection when caring for someone with Mpox and should follow safe protocols for handling test samples or sharp objects to avoid exposure.
11. Pre‐exposure vaccination is required for individuals who are at a higher risk of contracting it, such as medical personnel, laboratory personnel, scientists, reaction teams, healthcare workers, and captive animals to prevent the transmission of infection.
12. Get vaccinated within 4 days of being exposed to someone with Mpox for the best chance at preventing it.
13. Avoid high‐risk activities (like close contact or attending crowded events) if you haven't had both doses of the vaccine.
14. Enlighten the public on infection risks, ways to avoid getting sick, and potential treatments.

For infected people:

1. Stay home in a well‐ventilated room, and isolate yourself until the symptoms subside and lesions heal.
2. Wash your hands often—especially after touching sores or anything that might have been contaminated.
3. Wear a mask and cover up lesions when you're around others to reduce the risk of spreading the virus.
4. Disinfect shared spaces regularly and avoid touching shared items like dishes or towels.
5. Try saltwater rinses for mouth sores, or take baths with Epsom salts or baking soda to soothe your body sores.
6. Take pain relief like paracetamol or ibuprofen if needed.
7. Avoid Popping blisters or scratching sores as these actions have the potential to transmit the rash to other parts of the body, prolonging healing, and resulting in secondary infections.
8. Avoid shaving areas with sores until the scabs have fallen off and new skin has formed underneath.

The World Health Organization (WHO) and other international health organizations are still keeping an eye on the issue and offering guidance on Mpox control initiatives.

## 7 Impact of Mpox on South Asia

### 7.1 Public Perception and Behavior

After the 2022 Mpox outbreak, many low‐ and middle‐income countries in Southeast Asia began preparing for possible future outbreaks. Several countries have strengthened screening for fever and Mpox symptoms at airports and seaports. In India and Pakistan, high‐level government meetings have been held to assess national preparedness. As of August 22, 2024, India reported no active Mpox cases, and testing facilities have been expanded to around 32 centers. In Bangladesh, the Directorate General of Health Services has introduced a hotline for reporting suspected Mpox cases. Some health experts in India have criticized the WHO announcement, stating that it caused unnecessary public fear and that efforts should focus more on awareness among high‐risk groups, such as people living with HIV. Effective and timely actions by health ministries can help reduce panic and ensure better public communication. Kerala, the first Indian state to report both COVID‐19 and Mpox cases, demonstrated effective outbreak management by prioritizing active surveillance over fear‐based messaging, which helped maintain public confidence [60].

Bangladesh faces several challenges in managing Mpox, including limited ICU capacity, weak healthcare infrastructure, shortage of oxygen and ventilators, inadequate antiviral availability, and poor awareness of hygiene practices. High population density and limited medical knowledge further increase the risk of outbreaks. In the South Asian region, primary healthcare services are provided by both public and private sectors. The public sector mainly focuses on preventive and promotive care, while most curative services are delivered by private providers. However, immunization coverage in the private sector remains low, with only 25% of providers in Nepal and 7% in Bangladesh offering vaccination services [61].

Therefore, increasing healthcare capacity and vaccination coverage is essential to reducing the impact. The nation should prioritize implementing travel limits, encouraging safety precautions, investing in variant‐specific treatments, and improving testing, sanitation, and public awareness [62]. Primary healthcare (PHC) funding in South Asia is lower than planned in national health policies. Although India aims to spend two‐thirds of its health budget on PHC, only about 55% is currently allocated. The region also faces a shortage of healthcare workers, with only 3–17 workers per 10,000 people, far below the WHO recommendation. Most healthcare staff are concentrated in urban areas, leaving rural regions underserved. Although South Asian countries are adopting digital health and e‐health systems, effective data use remains limited due to poor data quality, lack of information from private healthcare providers, weak infrastructure, low digital skills among health workers, and fragmented data systems [61].

### 7.2 Economic and Social Impacts

Considering that 36% of the world's population lives in Bangladesh, Myanmar, Bhutan, Nepal, Sri Lanka, and China, the expansion of MPXV in India could increase the disease's global impact. A decline in tourism in the southeast region may occur, leading to a negative impact on the local economy. For a nation like Sri Lanka, which has already been declared bankrupt, this might prove to be more damaging [60].

Examining the COVID‐19 pandemic, we can observe a decline in the South Asian economy. Lockdowns, travel restrictions, quarantines, etc, imposed to contain the spread of the disease, resulted in multiple sectors shutting down, especially those requiring close human interaction. This caused massive layoffs throughout the region, which in turn forced people to cut down their consumption, further decreasing the demand for goods [63].

According to International Monetary Fund (IMF) data, the GDP per capita of South Asia in 2019 stood at US$2.01 thousand. However, during COVID‐19 in 2020, it declined to US$1.9 thousand. In the same period, the region's real GDP growth dropped sharply from 4.1% in 2019 to −4.5% in 2020 [64].

If the MPXV outbreak were to spread on a scale comparable to COVID‐19, it could potentially lead to similar economic consequences.

## 8 Future Threats and Preparedness

### 8.1 Potential of Mpox to Become a Pandemic

In recent years, outbreaks of infectious diseases are becoming increasingly common. Over the past 20 years, numerous epidemics and two global pandemic (swine flu and COVID‐19) outbreaks have emerged; significantly impacting the global economy, society, and public health [65]. After COVID‐19, the recent outbreaks of Mpox have us questioning whether this epidemic can turn into a new global pandemic.

MPXV is a double‐stranded DNA virus which means it has a slower rate of mutation. On the other hand, SARS‐CoV‐2 is an RNA virus that is more contagious, with a faster mutation rate [66, 67]. Both SARS‐CoV‐2 and H1N1 viruses spread via respiratory droplets and aerosols. On the contrary, MPXV is not airborne and spreads through direct contact with the infected individuals; making it less likely to infect a large population quickly [65]. There were initially no vaccines for COVID‐19; however, stockpiled smallpox vaccines can be effective against Mpox and aid in breaking the transmission chain [67]. Therefore, if we were to compare it with the most recent pandemic, Mpox has a low chance of developing into a pandemic, especially if precautionary measures are taken.

### 8.2 Preparedness of South Asian Countries

Although South Asia has a lower number of cases of Mpox compared to many parts of the world, South Asian countries must still take necessary measures to prevent the spread of Mpox. MPXV thrives in temperatures of 14°C–26°C and humidity of 65%–83%, which is close to the climate in India and many countries nearby [68]. There has been a shift in human behavior resulting from the easing of COVID restrictions, the return of international travel, large‐scale gatherings, and a rise in homosexual activities. These factors can result in a higher rate of transmission [69].

India, Bangladesh, Maldives, Pakistan, Sri Lanka, Bhutan, Afghanistan, and Nepal‐ all have Mpox diagnostic kits; although some of these countries do not have them in adequate amounts [70, 71, 72, 73, 74, 75, 76, 77]. In order to stop the spread of MPXV, the Taliban‐led interim government of Afghanistan has already requested international aid and increased surveillance [77, 78]. However, the underdeveloped healthcare system and limited access to healthcare, especially for women, are some challenges that the people of Afghanistan face [77, 79]. Following the increasing cases in India, the level of precaution has also been increased as health teams at entry points and airports have been briefed and trained about the situation. Moreover, a video conference conducted by the Directorate of Health Services (DGHS) with more than 200 participants highlighted the significance of strengthening surveillance and prompt case detection [36]. Even after taking such steps, India would still face difficulties in controlling the virus, if there were to be any wide‐scale outbreak, due to poor infrastructure [77]. Nepal would have difficulty battling future outbreaks due to a lack of healthcare facilities, low literacy rates, poor sanitation, and very few diagnostic laboratories [71]. However, hotline numbers have been launched in both Nepal (1115) and Bangladesh (16,263 or 10,655) in case of Mpox symptoms [71, 80]. Although there have been zero cases so far in Bangladesh, proactive measures have been implemented in Dhaka's airport, including 24/7 health desks, temperature scanners, escorting suspected patients to designated hospitals safely, and more. However, Bangladesh still does not have a strong surveillance system and needs better diagnostic laboratories [72]. The Maldives has also started preparations by training healthcare professionals and strengthening surveillance [74]. Pakistan has activated the National Command and Operation Center (NCOC) and International Health Regulations (IHR) committees and has also implemented several strategies to contain the spread of Mpox including tighter surveillance at entry points, improved laboratory networks, the establishment of quarantine facilities, distribution of education material to raise public awareness [70]. Meanwhile, the Health Emergency Operation Center (HEOC) and Technical Advisory Group of Bhutan have been activated. Visitors from affected countries are asked to self‐report any symptoms. Moreover, a formal request for Mpox ‐specific detection kits has been submitted to WHO and three vaccines for Mpox are available in the country at the moment [75]. Many South Asian countries have a social stigma associated with this disease which is why a lot of cases go unreported [71, 79]. Even though the countries are preparing more and more every day, steps need to be taken to dissipate this social stigma; otherwise, it would be impossible to prevent the spread of Mpox, and surveillance would also fail.

### 8.3 Measures to Mitigate Future Outbreaks in South Asia

LMICs have a shortage of trained personnel and national infection prevention and control program in healthcare settings to conduct surveillance activities and provide feedback. Moreover, manual data collection is strenuous and impractical. Limited laboratory facilities also contribute to difficulty in surveillance. Integrated surveillance systems should be established in all countries with the use of modern technology to enable rapid detection, outbreak control, and implementation of infection prevention measures in community settings. It is also crucial to strengthen the healthcare capacity of South Asian countries and to employ adequate healthcare workers. Additionally, laboratory capacities must also be increased [81].

### 8.4 Comparative Analysis: Lessons Learnt From COVID‐19

Applying lessons from the COVID‐19 pandemic is important for controlling Mpox. Early detection, proper surveillance, and quick response can help prevent outbreaks. Countries that used strong testing and contact‐tracing systems during COVID‐19 were able to reduce transmission, and similar approaches can be used for Mpox. The COVID‐19 pandemic also revealed major inequalities in vaccine distribution, especially in low‐income countries. For Mpox, fair access can be improved by promoting local production of vaccines and diagnostic tools to reduce dependence on imports. Community involvement is equally important. During COVID‐19, misinformation slowed public health responses, while community‐based programs helped spread accurate information. Involving local leaders, healthcare workers, and community organizations can improve public awareness and acceptance of preventive measures. International cooperation in vaccine development and distribution, as seen with organizations like GAVI and CEPI, is also essential for effective Mpox control [82]. One of the social impacts of COVID‐19 pandemic was blame and racism against people of Asian descent, globally. The media and key opinion leaders aggravated the stigma by initial naming of COVID‐19 virus as the “Wuhan” or “China” virus. The population mostly affected by Mpox resides in Central and West Africa. It is said to have spread to North America or Europe by the movement of people or animals from these areas. The two strains of Mpox are labeled “West African” and “Congo Basin Central,” after the name of these regions. Thus, the World Health Organization (WHO) recently renamed the virus from monkeypox to Mpox. It also plans to rename the clades, in order to reduce the potential for stigma [83].

One of the major stigmas attached to Mpox is regarding men who have sex with men (MSM). This stigma may worsen false information about the illness and discourage people from getting tested or receiving treatment. Public health campaigns must use inclusive, non‐stigmatizing language that highlights the risk of infection for everyone in order to combat this [82].

### 8.5 One Health Perspective on Mpox Surveillance and Zoonotic Spillover in South Asia

One Health approaches are vital for Mpox, integrating human, animal, and environmental monitoring to address gaps in early detection of orthopoxviruses. Sri Lanka integrates Mpox into existing frameworks but lacks robust veterinary‐environmental links, as seen in regional prioritization workshops. Multisectoral tools like Joint Risk Assessments strengthen collaboration in South‐East Asia [84]. Close human‐animal contacts in South Asia, via livestock, wildlife trade, and deforestation, heighten Mpox spillover, with hotspots in eastern Himalayas and Indonesia‐Malaysia overlapping low‐vaccination areas. Orthopoxvirus genomic features predict reservoirs, urging wildlife monitoring. Illegal trade amplifies risks across the region [85].

## 9 Limitations and Future Research

As discussed earlier, South Asia which mostly constitutes of LMICs, does not have a strong surveillance system and diagnostic facilities; resulting in underreporting or delayed reporting. In rural or remote regions of South Asia, there are hardly any proper hospitals where people can be treated. Moreover, due to social stigma, many people refuse to disclose their condition and deny receiving treatment from actual hospitals. Therefore, the actual number of cases and transmission dynamics might not be accurate.

In this region, there is a lack of studies regarding the use of antivirals and its efficacy in Mpox. More South‐Asia based research and survey must be conducted, not only for Mpox, but also various other viral diseases to identify the trend in transmission so that appropriate steps can be taken when necessary. Since South Asia is so densely populated, any major outbreak in this region can raise massive public health concerns. Thus, research must also be done on ways the surveillance and healthcare system can be strengthened. Research regarding which enzootic virus has the potential to become zoonotic might be helpful in preventing any future outbreaks. Genomic studies for surveillance might also be helpful for future preparedness.

## 10 Conclusion

Mpox, previously endemic to Africa, has spread globally and affected South Asian countries such as India, Pakistan, Nepal, and Sri Lanka. Although case numbers remain relatively low, high population density, limited healthcare infrastructure, and social stigma heighten the risk of rapid transmission and underreporting. Preventive efforts such as screening at entry points, awareness campaigns, and vaccination initiatives have been introduced; however, inadequate diagnostic capacity and critical care facilities remain major challenges. Clinically, antivirals such as Tecovirimat, Cidofovir, and Brincidofovir are being used, but emerging Tecovirimat resistance and nephrotoxicity risks with Cidofovir demand cautious administration and close monitoring. Vaccination continues to be the most effective preventive strategy, with JYNNEOS favored for immunocompromised individuals and ACAM2000 for those without contraindications. Despite this, vaccine coverage among high‐risk groups including healthcare workers and men who have sex with men remains insufficient.

To improve preparedness, South Asian healthcare systems must adopt region‐specific strategies. These include expanding vaccine accessibility through targeted outreach programs, ensuring post‐exposure prophylaxis within 4 days of exposure, and maintaining adequate stockpiles of antivirals and immunoglobulins. Standardized clinical protocols for antiviral use and drug‐resistance surveillance are essential. Simultaneously, integrated digital surveillance systems and stigma‐reduction campaigns should be prioritized to encourage early case reporting. Strengthening diagnostic laboratories, increasing ICU capacity, and training frontline workers are critical to sustaining outbreak control and minimizing public health impact.

## Author Contributions

**Jannatul Mawa:** writing – review and editing, writing – original draft. **Shariun Nahar Rimun:** writing – review and editing, writing – original draft. **Nujhat Zayma Rahman:** writing – review and editing, writing – original draft. **Mohiuddin Ahmed Bhuiyan:** visualization, writing – review and editing. **Mohammad Shahriar:** visualization, writing – review and editing. **Ramisa Anjum:** conceptualisation, supervision.

## Ethics Statement

The authors have nothing to report.

## Conflicts of Interest

The authors declare no conflicts of interest.

## Transparency Statement

The lead author Ramisa Anjum affirms that this manuscript is an honest, accurate, and transparent account of the study being reported; that no important aspects of the study have been omitted; and that any discrepancies from the study as planned (and, if relevant, registered) have been explained.

## Data Availability

Data sharing not applicable to this article as no datasets were generated or analyzed during the current study. All authors have read and approved the final version of the manuscript. Ramisa Anjum had full access to all of the data in this study and takes complete responsibility for the integrity of the data and the accuracy of the data analysis The authors confirm that the data supporting the findings of this study are available within the article [and/or] its supporting materials.
